# Genetic polymorphisms of *N*-acetyltransferase 1 and 2 and risk of cigarette smoking-related bladder cancer

**DOI:** 10.1038/sj.bjc.6690727

**Published:** 1999-10

**Authors:** F-I Hsieh, Y-S Pu, H-D Chern, L-I Hsu, H-Y Chiou, C-J Chen

**Affiliations:** Graduate Institute of Epidemiology, College of Public Health, 1 Jen-Ai Road Section 1; Department of Oncology, Institute of Pharmaceutical Sciences, College of Medicine, National Taiwan University; Department of Public Health, School of Medicine, Taipei Medical College, Taipei, Taiwan

**Keywords:** *N*-acetyltransferase 1, *N*-acetyltransferase 2, cigarette smoking, bladder cancer

## Abstract

Aromatic amines from cigarette smoking or occupational exposure, recognized risk factors for bladder cancer, are metabolized by *N*-acetyltransferases (NAT). This study examined the association of (NAT) 1 and 2 genotypes with the risk of smoking-related bladder cancer. A total of 74 pathologically confirmed bladder cancer patients and 184 controls were serially recruited from the National Taiwan University Hospital. History of cigarette smoking and other risk factors for bladder cancer was obtained through standardized questionnaire interview. Peripheral blood lymphocytes were collected from each subject and genotyped for NAT1 and NAT2 by DNA sequencing and polymerase chain reaction-restriction fragment length polymorphism methods. Allele frequency distributions of NAT1 and NAT2 were similar between cases and controls. There was a significant dose–response relationship between the risk of bladder cancer and the quantity and duration of cigarette smoking. The biological gradients were significant among subjects carrying NAT1*10 allele or NAT2 slow acetylators, but not among NAT2 rapid acetylators without NAT1*10 allele. The results are consistent with the hypothesis that NAT1 and NAT2 might modulate the susceptibility to bladder cancer associated with cigarette smoking. © 1999 Cancer Research Campaign


*British Journal of Cancer* (1999) **81**(3), 537–541
© 1999 Cancer Research Campaign
Article no. bjoc.1999.0727

### Genetic polymorphisms of *N*-acetyltransferase 1 and 2 and risk of cigarette smoking-related bladder cancer

**F-I Hsieh[1], Y-S Pu[2], H-D Chern[3], L-I Hsu[1], H-Y Chiou[4] and C-J Chen[1]**

[1]Graduate Institute of Epidemiology, College of Public Health, 1 Jen-Ai Road Section 1, [2]Department of Oncology, [3]Institute of Pharmaceutical Sciences, College of Medicine, National Taiwan University, [4]Department of Public Health, School of Medicine, Taipei Medical College, Taipei, Taiwan

**Summary** Aromatic amines from cigarette smoking or occupational exposure, recognized risk factors for bladder cancer, are metabolized by *N*-acetyltransferases (NAT). This study examined the association of (NAT) 1 and 2 genotypes with the risk of smoking-related bladder cancer. A total of 74 pathologically confirmed bladder cancer patients and 184 controls were serially recruited from the National Taiwan University Hospital. History of cigarette smoking and other risk factors for bladder cancer was obtained through standardized questionnaire interview. Peripheral blood lymphocytes were collected from each subject and genotyped for NAT1 and NAT2 by DNA sequencing and polymerase chain reaction-restriction fragment length polymorphism methods. Allele frequency distributions of NAT1 and NAT2 were similar between cases and controls. There was a significant dose–response relationship between the risk of bladder cancer and the quantity and duration of cigarette smoking. The biological gradients were significant among subjects carrying NAT1*10 allele or NAT2 slow acetylators, but not among NAT2 rapid acetylators without NAT1*10 allele. The results are consistent with the hypothesis that NAT1 and NAT2 might modulate the susceptibility to bladder cancer associated with cigarette smoking. © 1999 Cancer Research Campaign

**Keywords:** *N*-acetyltransferase 1; *N*-acetyltransferase 2; cigarette smoking; bladder cancer

Cigarette smoking is an important risk factor for bladder cancer (Cluade et al, 1986; Jensen et al, 1987; Augustine et al, 1988; Schairer et al, 1988; Burch et al, 1989; Lopez-Abente et al, 1991). Cigarette smoke includes 2-naphthylamine and 4-aminobiphenyl, which are recognized as human and animal carcinogens (International Agency for Research on Cancer, 1986).

The *N*-acetyltransferase (NAT) has two isozymes, NAT1 and NAT2. Each gene is located on chromosome 8 and has an open reading frame of 870-base pair. The enzyme activities of NAT1 and NAT2 are polymorphic (Grant et al, 1992; Weber and Vatsis, 1993; Bell et al, 1995*a*). NAT1 gene has sequence polymorphism in the 3′ untranslated region, the known alleles include NAT1*4 (wild-type), NAT1*10, NAT1*11 and NAT1*3 (Vatsis and Weber, 1993). NAT2 gene has a number of point mutations, which result in decreased NAT activity. The four mutations are NAT2*5 (C at nucleotide 341), NAT2*6 (A at nucleotide 590), NAT2*7 (A at nucleotide 857) and NAT2*14 (A at nucleotide 191) (Vatsis et al, 1995). NAT1*10 allele may increase enzyme activity and two NAT2 mutant alleles may decrease enzyme activity (Hickman and Sim, 1991; Vineis et al, 1994; Bell et al, 1995*a*). Both NAT1 and NAT2 enzymes catalyse the *N*-acetylation of aromatic amine which is considered a detoxifying process. NAT1 also catalyses the *O*-acetylation forming *N*-hydroxyarylamines and converts arylhydroxamic acids into mutagenic acetoxy esters by *N*,*O*-acetyltransferase reaction (Hein et al, 1992; Bartsch et al, 1993; Skipper and Tannenbaum, 1994).

While NAT2 slow acetylators were found to have an increased risk of bladder cancer among cigarette smokers (Risch et al, 1995; Brockmoller et al, 1996), there were inconsistent findings regarding the effect of NAT1 genetic polymorphism on bladder cancer (Okkels et al, 1997; Taylor et al, 1998). The specific aim of this study is to examine the effects of NAT1 and NAT2 on cigarette smoking-related bladder cancer through a case control comparison design.

*Received 19 November 1998*
*Revised 21 February 1999*
*Accepted 2 March 1999*

*Correspondence to:* C-J Chen

### SUBJECTS AND METHODS

### Study subjects

In this case control study, 74 patients affected with urinary bladder cancer were serially recruited from National Taiwan University Hospital. All of them were diagnosed histologically to be affected with transitional cell carcinoma. A total of 184 control subjects within the same age range of cases were recruited from health examination clinic (77.8%) and urology clinic (22.2%) in the same hospital. They were not affected with any cancer. Subjects who had lived in the arseniasis-endemic area were excluded from this study. The proportion of males was 78.4% for cases and 77.7% for controls. Cases and controls had similar frequency for distribution of age. There were 44.6% cases and 54.3% controls with an age below 65 years old. Because there were insufficient male elderly subjects to be selected as controls from the health examination clinic, several male controls were recruited from the urology clinic among patients with benign prostatic hypertrophy. Benign prostatic hypertrophy is a very common condition among elderly males in Taiwan, and no association between NAT and benign prostatic hypertrophy has ever been documented.

Standardized personal interview based on a structured questionnaire was carried out to collect information on risk factors, including sociodemographic characteristics, residential and occupational history, habits of cigarette smoking and consumption of alcohol, tea and coffee, dietary habits, personal and family history of urinary disease and cancers. Duration and quantity of cigarette smoking was inquired in detail. Subjects who had smoked cigarettes more than 3 days a week for more than 6 months were classified as cigarette smokers and the others as non-smokers. A peripheral blood sample was collected from each subject using disposable vacuum syringe containing heparin. DNA was extracted from peripheral lymphocytes by Genomix DNA extraction kit (Talent, Follatoio, Italy) resuspended in deionized distilled water, and stored at –20°C until genotyping.

### Genotypes of NAT1 and NAT2

NAT1 was amplified by polymerase chain reaction (PCR) using the primers N1-OA (5′-GCTCACCAGTTATCAACTGAC) and N1-OB (5′-AACCAACATTAAAAGCTTTCT) and resulted in a 233-base pair amplificate. The PCR mixture was composed of 1000–2000 ng DNA, 20 pmole of each NAT1 primers, 1 U *Taq* polymerase (Takara Taq, Takara Shuzo, Japan), 5 µl 10 × PCR buffer (100 mM Tris–HCl (pH 8.3), 500 mM potassium chloride (KCl), 15 mM magnesium chloride ($MgCl_2$)) and 4 µl 2.5 mM deoxynucleoside triphosphates in a final volume of 50 µl. The reaction mixture was placed for 5 min at 94°C, and then subjected to 30 cycles of 94°C for 30 s, 52°C for 30 s and 72°C for 45 s. This was followed by a final step at 72°C for 10 min. Negative control was included in each set of PCR analyses. The PCR products were electrophoresed in 1.5% agarose gel (FMC, Rockland, ME, USA) and then extracted the DNA by QIAquick gel extraction kit (QIAGEN, Germany). The product was processed with a Taq-dye terminator cycle sequencing ready reaction kit (Applied Biosystems Inc., Foster City, CA, USA). The fragments were then analysed with an Applied Biosystems 373A automated sequencer with a denaturing 6% polyacrylamide gel.

For NAT2, a PCR was carried out with PCR primers N5 (5′-GGAACAAATTGGACTTGG) and N4 (5′-TCTAGCAT-GAATCACTCTGC). The PCR mixture was composed of 1000–2000 ng DNA, 50 pmol of each primer, 5 U *Taq* polymerase (Takara Taq, Takara Shuzo, Japan), 5 µl 10 × PCR buffer 100 mM Tris–HCl (pH 8.3), 500 mM KCl, 15 mM $MgCl_2$ and 4 µl 2.5 mM deoxynucleoside triphosphates in a final volume of 50 µl. It was denatured at 94°C for 5 min and subjected to 35 cycles of 94°C for 30 s, 52°C for 30 s and 72°C for 90 s. A final 72°C extension for 10 min was performed. The whole intronless NAT2 gene was resulted in a 1093-base pair amplificate. It was then digested with 5 U *Kpn*I (AGS Gmbh, Germany) for NAT2*5 fragment, 10 U *Bam*HI (AGS Gmbh, Germany) for NAT2*7 fragment and 5 U *Alu*I/*Msp*I (Boehringer Mannheim, Germany) for NAT2*14 fragment at 37°C overnight, respectively, and 5 U *Taq*I (Boehringer Mannheim, Germany) for NAT2*6 at 65°C overnight. The NAT2*5, NAT2*7 and NAT2*14 fragments were separated in 2% agarose gel, and NAT2*6 fragments in 3% agarose gel. Individuals with two mutant alleles including NAT2*5, NAT2*6, NAT2*7 or NAT2*14 (Vatsis et al, 1995) were classified as slow acetylators and the others were rapid acetylators.

### Statistical analysis

The differences in frequency distributions of bladder cancer risk factors between cases and controls were tested for their statistical significance by $\chi^2$ tests. In the univariate analysis, odds ratios (OR) with a 95% confidence interval (CI) for each risk factor were calculated after the adjustment for age (continuous variable), sex and educational level (categorized variable) through logistic regression analysis. Cases had a slightly older mean age at recruitment, and lower educational levels than controls.

### RESULTS

While fewer cases (10%) than controls (20%) were habitual coffee drinkers, there was no significant association between coffee drinking and bladder cancer after adjustment for age, sex and educational level (OR = 0.6, 95% CI = 0.2–1.4). The proportions of habitual alcohol drinking were 30% for cases and 24% for controls, a multivariate-adjusted OR of 1.1 (95% CI 0.6–2.2). Forty-five per cent cases and 48% controls were tea drinkers with a multivariate-adjusted OR of 0.9 (95% CI 0.5–1.7). Few study subjects had well documented occupational exposures related to bladder cancer. Only one case and one control were engaged in printing and painting industry, and three cases and two controls were professional drivers. The consumption of coffee, alcohol and tea, as well as the above occupational exposures, were not considered as confounding factors.

Table 1 compares the cigarette smoking habit between bladder cancer cases and healthy controls. Cases had a higher percentage of cigarette smokers than controls ($0.05 < P < 0.10$) showing an OR of developing bladder cancer of 1.90 (95% CI 0.99–3.64) after

**Table 1** Comparison of cigarette smoking habit between bladder cancer cases and healthy controls

| | | Cases | | Controls | | Adjusted odds ratio[a] |
|---|---|---|---|---|---|---|
| Cigarette smoking | Group | No. | (%) | No. | (%) | (95% CI) |
| Habit | No | 35 | (47.3) | 119 | (64.7) | 1.00 (referent) |
| | Yes | 39 | (52.7) | 65 | (35.3) | 1.91 (0.99–3.64)[d] |
| Duration (year)[b] | 0 | 35 | (49.3) | 119 | (66.5) | 1.00 (referent) |
| | <33 | 12 | (16.9) | 27 | (15.1) | 1.41 (0.58–3.42) |
| | ≥33 | 24 | (33.8) | 33 | (18.4) | 2.52 (1.16–5.50)[c] |
| Quantity (packs per day)[b] | 0 | 35 | (47.9) | 119 | (65.7) | 1.00 (referent) |
| | <1 | 18 | (24.7) | 36 | (19.9) | 1.76 (0.82–3.78) |
| | ≥1 | 20 | (27.4) | 26 | (14.4) | 2.35 (1.05–5.24)[c] |

[a]Adjustment for age, sex and educational level through logistic regression analysis. [b]Duration and/or quantity of cigarette consumption were not available for three cases and six controls [c]$P < 0.05$ bases on the significance test of the odds ratio. [d]$0.05 < P < 0.10$ bases on the significance test of the odds ratio. [e]$P < 0.05$ bases on the trend test. CI: confidence interval.

© 1999 Cancer Research Campaign

**Table 2** Comparison of NAT 1 and NAT 2 allele and genotype frequency between cases of bladder cancer and controls

| Gene | Allele/Genotype | Cases No. | Cases (%) | Controls No. | Controls (%) | *P*-value[a] |
|---|---|---|---|---|---|---|
| NAT1[b] | *3 | 4 | (3.1) | 11 | (3.2) | 0.92 |
| | *4 | 75 | (57.7) | 186 | (54.4) | |
| | *10 | 50 | (38.5) | 143 | (41.8) | |
| | *11 | 1 | (0.7) | 2 | (0.6) | |
| NAT2[b] | NAT2*4 (wild) | 85 | (58.2) | 192 | (52.6) | 0.70 |
| | NAT2*5 | 6 | (4.1) | 22 | (6.0) | |
| | NAT2*6 | 33 | (22.6) | 99 | (27.0) | |
| | NAT2*7 | 21 | (14.4) | 51 | (13.9) | |
| | NAT2*14 | 1 | (0.7) | 2 | (0.5) | |
| NAT1 | Without *10 | 25 | (38.5) | 60 | (35.1) | 0.63 |
| | With *10 | 40 | (61.5) | 111 | (64.9) | |
| NAT2 | Slow acetylator | 15 | (20.6) | 44 | (24.0) | 0.55 |
| | Rapid acetylator | 58 | (79.4) | 139 | (76.0) | |
| NAT1/NAT2 | Without *10/slow | 6 | (9.4) | 18 | (10.6) | 0.53 |
| | Without *10/rapid | 18 | (28.1) | 42 | (24.7) | |
| | With *10/slow | 5 | (7.8) | 25 | (14.7) | |
| | With *10/rapid | 35 | (54.7) | 85 | (50.0) | |

[a]Based on $\chi^2$ test. [b]NAT1 genotypes were not available for nine cases and 13 controls, while NAT2 genotypes were not available for one case and one control.

**Table 3** Association between cigarette smoking and bladder cancer risk stratified by genotypes of NAT1 and NAT2

| Cigarette smoking | Group | Adjusted odds ratio (95% confidence interval)[a]: NAT2 rapid acetylator Without NAT1 *10 | Adjusted odds ratio (95% confidence interval)[a]: With NAT1 *10 or NAT2 slow acetylator |
|---|---|---|---|
| Habit | No | 1.00 (referent) | 1.00 (referent) |
| | Yes | 2.07 (0.32–13.30) | 2.34 (1.03–5.31)[b] |
| Duration (year) | 0 | 1.00 (referent) | 1.00 (referent) |
| | <33 | 0.55 (0.04–7.83) | 1.78 (0.58–5.45)[b] |
| | ≥33 | 4.33 (0.56–33.41) | 3.08 (1.14–8.32)[b,c] |
| Quantity (pack/day) | 0 | 1.00 (referent) | 1.00 (referent) |
| | <1 | 2.65 (0.34–20.70) | 2.13 (0.82–5.53) |
| | ≥1 | 1.49 (0.16–13.93) | 3.22 (1.17–8.86)[b,c] |

[a]Adjustment for age, sex and educational level through logistic regression analysis. [b]$P < 0.05$ based on the significance test of the odds ratio. [c]$P < 0.05$ based on the trend test.

adjustment for age, sex and educational level. There were significant dose–response relationships between the risk of bladder cancer and the duration and quantity of cigarette smoking.

The frequency distributions of NAT1 and NAT2 alleles and genotypes in cases and controls are compared in Table 2. Cases and controls had similar allele frequency distributions of NAT1 and NAT2. A total of 61.5% cases and 64.9% controls had one or two alleles of NAT1*10; 20.6% cases and 24.0% controls were NAT2 slow acetylators. The genotype frequency distributions of NAT1 and NAT2 were alike in cases and controls. The combined frequency distribution of NAT1 and NAT2 was also similar in cases and controls.

As shown in Table 3, the OR of developing bladder cancer for cigarette smoking was further analysed by stratifying study subjects according to NAT1 and NAT2 genotypes. Among cases, those subjects carrying NAT1*10 allele and NAT2 slow acetylators had an increased bladder cancer risk associated with cigarette smoking, they were classified as one group to be compared with cases with NAT2 rapid acetylators and without NAT1*10 allele as another group. A significant association between bladder cancer and cigarette smoking was observed among subjects carrying NAT1*10 allele or NAT2 slow acetylators showing an OR of 2.34 (95% CI 1.03–5.31), but not among NAT2 rapid acetylators without NAT1*10 allele (OR 2.07, 95% CI 0.32–13.30). The dose–response relationships between the risk of bladder cancer and the quantity and duration of cigarette smoking were also statistically significant among those who had NAT1*10 allele or NAT2 slow acetylators, but not for cases with NAT2 rapid acetylators but without NAT1*10 allele.

### DISCUSSION

As in previous studies of cigarette smoking and bladder cancer, we also observed an increased risk of bladder cancer among cigarette smokers in this study. The metabolism of carcinogenic arylamines in tobacco smoke is mediated by enzymes including NAT1 and NAT2. Both NAT1 and NAT2 are genotypically and phenotypically polymorphic with variable genotype frequencies in

© 1999 Cancer Research Campaign different ethnic groups. The allele frequency of NAT1*10 in controls of this study was 41.8%, which is significantly higher ($\chi^2 = 4.00$, $P < 0.05$) than the 30.0% observed in Caucasians (Bell et al, 1995*b*). The frequency of NAT2 slow acetylator in controls of this study was 24.0%, which is similar to that observed among Chinese in Hong Kong (27.0%) (Lin et al, 1993) and lower than that among American Caucasians (55.0%) (Bell et al, 1993). The percentage of NAT2 slow acetylator is significantly lower in Taiwanese than in American Caucasians ($\chi^2 = 20.14$, $P < 0.001$).

NAT2 slow acetylators were found to have an increased risk of bladder cancer among cigarette smokers (Risch et al, 1995; Brockmoller et al, 1996). The NAT1*10 allele alone was reported to be a risk factor in one study (Taylor et al, 1998), but not in another (Okkels et al, 1997). In this study, the genetic polymorphism of neither NAT1 nor NAT2 was significantly associated with the development of bladder cancer. However, the significant dose–response relationships between cigarette smoking and bladder cancer were observed in this study among those with NAT1*10 or NAT2 slow acetylators, but not among NAT2 rapid acetylators without NAT1*10 allele.

Some aromatic amines are metabolically inactivated through acetylation in the liver mainly by NAT2 (Kadlubar and Badawi, 1995). Rapid NAT2 acetylators are considered to have a higher level of non-toxic metabolites and a lower risk of bladder cancer than slow NAT2 acetylators. Some other arylamines were catalysed by cytochrome P450 1A2 in the liver, and the *N*-hydroxy arylamine metabolites can then enter the blood stream. The *N*-hydroxy arylamine metabolites are further metabolized into highly electrophilic *N*-acetoxy derivatives by NAT1, which is expressed mainly in urinary bladder epithelium (Kirlin et al, 1989; Frederickson et al, 1994). This will lead to an elevated level of arylamine-DNA adducts and an increased risk of bladder cancer. The dose–response relationship between cigarette smoking and bladder cancer is thus significant among cigarette smokers who have NAT1*10 allele and/or cigarette-smoking NAT2 slow acetylators.

In addition to NAT1 and NAT2, the metabolism of arylamines also involves a number of other enzymes including sulphotransferases (Kato and Yamazoe, 1994), prostaglandin H synthase (Smith et al, 1992), cytochrome P450 1A2 (Butler et al, 1989; Fleming et al, 1994) and glucuronyltransferases. These enzymes may also contribute to the metabolic steps of arylamines relevant to the development of bladder cancer. Further examination of effects of these enzymes on the cigarette smoking-related bladder cancer in humans is noteworthy.

### ACKNOWLEDGEMENTS

This study was supported by grants from the National Sciences Council (NSC-86-2314-B-002-336, NSC-87-2314-B-002-004, NSC-88-2314-B-002-005) and National Health Research Institutes, Department of Health, Executive Yuan (DOH-86-HR-503, DOH-87-HR-503, DOH-88-HR-503), Republic of China.

### REFERENCES

Augustine A, Hebert JR, Kabat GC and Wynder EL (1988) Bladder cancer in relation to cigarette smoking. *Cancer Res* **48**: 4405–4408

Bartsch H, Malaveille C, Friesen M, Kadlubar FF and Vineis P (1993) Black (ari-cured) and blond (flue-cured) tobacco cancer risk IV: molecular dosimetry studies implicate aromatic amines as bladder carcinogens. *Eur J Cancer* **29A**: 1199–1207.

Bell DA, Taylor JA, Butler MA, Stephens E, Wiest J, Brubaker L, Kadlubar FF and Lucier GW (1993) Genotype/phenotype discordance for human arylamine N-acetyltransferase (NAT2) reveals a new slow-acetylator allele common in African-Americans. *Carcinogenesis* **14**: 1689–1692

Bell DA, Badawi AF, Lang NP, Ilett KF, Kadlubar FF and Hirvonen A (1995*a*) Polymorphism in the N-acetyltransferase 1 (NAT1) polyadenylation signal: association of NAT1*10 allele with higher N-acetylation activity in bladder and colon tissue. *Cancer Res* **55**: 5226–5229.

Bell DA, Stephens EA, Castranio T, Umbach DM, Watson M, Deakin M, Elder J and Berkowitz RS (1995*b*) Polyadenylation polymorphism in the acetyltransferase 1 gene (NAT1) increases risk of colorectal cancer. *Cancer Res* **55**: 3537–3542

Brockmoller J, Cascorbi I, Kerb R and Roots I (1996) Combined analysis of inherited polymorphisms in arylamine N-acetytransferase 2, glutathione S-transferases M1 and T1, microsomal epoxide hydrolase, and cytochrome P450 enzymes as modulators of bladder cancer risk. *Cancer Res* **56**: 3915–3925.

Burch JD, Rohan TE, Howe GR, Risch HA, Hill GB, Steele R and Miller AB (1989) Risk of bladder cancer by source and type of tobacco exposure: a case-control study. *Int J Cancer* **44**: 626–628

Butler MA, Iwasaki M, Guengerich FP and Kadlubar FF (1989) Human cytochrome $P\text{-}450_{pa}$ (p4501A2), the phenacetin O-deethylase, is primarily responsible for the hepatic 3-demethylation of caffeine and N-oxidation of carcinogenic arylamines. *Proc Natl Acad Sci USA* **86**: 7696–7700

Chiou HY, Hsueh YM, Liaw KF, Horng SF, Chiang MH, Pu YS, Lin JS, Huang CH and Chen CJ (1995) Incidence of internal cancers and ingested inorganic arsenic: a seven-year follow-up study in Taiwan. *Cancer Res* **55**: 1296–1300

Cluade J, Kunze E, Frentzel-Beyme R, Paczkowski K, Schnetder J and Schubert H (1986) Life-style and occupational risk factors in cancer of the lower urinary tract. *Am J Epidemiol* **124**: 578–589.

Fleming CM, Persad R, Kaisary A, Smith P, Adedoyin A, Porter J, Wilkinson GR and Branch RA (1994) Low activity of dapsone N-hydroxylation as a susceptibility risk factor in aggressive bladder cancer. *Pharmacogenetics* **4**: 199–207

Frederickson SM, Messing EM, Resnikoff CA and Swaminathan S (1994) Relationship between in vivo acetylator phenotypes and cytosolic N-acetyltransferase and O-acetyltransferase activities in human uroepithelial cells. *Cancer Epidemiol Biomark Prev* **3**: 25–32

Grant DM, Vohra P, Avis Y and Ima A (1992) Detection of a new polymorphism of human arylamine N-acetyltransferase (NAT1) using p-aminosalicylic acid as an in vivo probe. *J Basic Clin Physiol Pharmacol* **3**: 244

Hein DW, Rustan TD, Doll MA, Bucher KD, Ferguson RJ, Feng Y, Furman EJ and Gray K (1992) Acetyltransferases and susceptibility to chemicals. *Toxicol Lett* **64/65**: 123–130

Hickman D and Sim E (1991) N-acetyltransferase polymorphism comparison of phenotype and genotype in humans. *Biochem Pharmacol* **42**: 1007–1014

International Agency for Research on Cancer (1986) *Tobacco Smoking. IARC Monographs on the Evaluation of Carcinogenic Risk of Chemicals to Humans*, Vol. **38**, pp. 35–394. International Agency for Research on Cancer: Lyon

Jensen OM, Wahrendorf J, Blettner M, Knudsen JB and Sorensen BL (1987) The Copenhagen case-control study of bladder cancer: role of smoking in invasive and non-invasive bladder tumours. *J Epidemiol Community Health* **41**: 30–36

Kadlubar FF and Badawi AF (1995) Genetic susceptibility and carcinogen-DNA adduct formation in human urinary bladder carcinogenesis. *Toxicol Lett* **82/83**: 627–632

Kato R and Yamazoe Y (1994) Metabolic activation of N-hydroxylated metabolites of carcinogenic and mutagenic arylamines and arylamides by esterification. *Drug Metabolism Rev* **26**: 413–430

Kirlin WG, Trinidad A, Yerokun T, Ogolla F, Ferguson RJ, Andrews AF, Brady PK and Hein DW (1989) Polymorphic expression of acetyl coenzyme A-dependent *O*-acetyltransferase-mediated activation of *N*-hydroxyarylamines by human bladder cytosols. *Cancer Res* **49**: 2448–2454

Lin HJ, Han CY, Lin BK and Hardy S (1993) Slow acetylator mutations in the human polymorphic *N*-acetyltransferase gene in 786 Asians, Blacks, Hispanics, and Whites: application to metabolic epidemiology. *Am J Hum Genet* **52**: 827–834

Lopez-Abente G, Gonzalez CA, Errezola M, Escolar A, Lzarzugaza I, Nebot M and Riboli E (1991) Tobacco smoke inhalation pattern, tobacco type, and bladder cancer in Spain. *Am J Epidemiol* **134**: 830–839

Okkels H, Sigsgaard T, Wolf H and Autrup H (1997) Arylamine N-acetyltransferase 1 (NAT1) and 2 (NAT2) polymorphisms in susceptibility to bladder cancer: the influence of smoking. *Cancer Epidemiol Biomark Prev* **6**: 225–231

 © 1999 Cancer Research Campaign

Risch A, Wallace DMA, Bathers S and Sim E (1995) Slow N-acetylation genotype is a susceptibility factor in occupational and smoking related bladder cancer. *Human Mol Genet* **4**: 231–236

Schairer C, Hartge P, Hoover RN and Silverman DT (1988) Racial differences in bladder cancer risk: a case-control study. *Am J Epidemiol* **128**: 1027–1037

Skipper PL and Tannenbaum SR (1994) Molecular dosimetry of aromatic amines in human populations. *Environ Health Perspect* **102**: 17–21

Smith BJ, Bebruin L, Josephy PD and Eling TE (1992) Mutagenic activation of benzidine requires prior bacterial acetylation and subsequent conversion by prostaglandin-H synthase to 4-nitro-4(acetylamino)biphenyl. *Chem Res Toxicol* **5**: 431–439

Taylor JA, Umbach DM, Stephens E, Castranio T, Paulson D, Robertson C, Mohler JL and Bell DA (1998) The role of N-acetylation polymorphisms at NAT1 and NAT2 in smoking-associated bladder cancer: evidence of a gene–gene–exposure three way interaction. *Cancer Res* **58**: 3603–3610

Vatsis KP and Weber WW (1993) Structural heterogeneity of Caucasian *N*-acetyltransferase at the NAT1 locus. *Arch Biochem Biophys* **301**: 71–76

Vatsis KP, Weber WW, Bell DA, Dupret JM, Evans DAP, Grant DM, Hein DW, Lin HJ, Meyer UA, Relling MV, Sim E, Suzuki T and Tamazoe Y (1995) Nomenclature for *N*-acetyltransferases. *Pharmacogenetics* **5**: 1–9

Vineis P, Bartsch H, Caporaso N, Harrington AM, Kadlubar FF, Landi MT, Malaveille C, Shields PG, Skipper P, Talaska G and Tannenbaum SR (1994) Genetically based *N*-acetyltransferase metabolic polymorphism and low-level environmental exposure to carcinogens. *Nature* **369**: 154–156

Weber WW and Vatsis KP (1993) Individual variability in p-aminobenzoic acid N-acetylation by human N-acetyltransferase (NATl) of peripheral blood. *Pharmacogenetics* **3**: 209–212

*© 1999 Cancer Research Campaign*
